# Cardiac MRI: An Overview of Physical Principles With Highlights of Clinical Applications and Technological Advancements

**DOI:** 10.7759/cureus.55519

**Published:** 2024-03-04

**Authors:** Mason T Stoltzfus, Matthew D Capodarco, FNU Anamika, Vasu Gupta, Rohit Jain

**Affiliations:** 1 Neurosurgery, Penn State College of Medicine, Hershey, USA; 2 Radiology, Penn State University College of Medicine, Milton S. Hershey Medical Center, Hershey, USA; 3 Internal Medicine, University College of Medical Sciences, New Delhi, IND; 4 Internal Medicine, Dayanand Medical College and Hospital, Ludhiana, IND; 5 Internal Medicine, Penn State University College of Medicine, Milton S. Hershey Medical Center, Hershey, USA

**Keywords:** physics of cardiac mri, radiology history, cost-utility analysis, artificial intelligence in radiology, cardiac imaging-mri

## Abstract

The purpose of this review is to serve as a concise learning tool for clinicians interested in quickly learning more about cardiac magnetic resonance imaging (CMR) and its physical principles. There is heavy coverage of the basic physical fundamentals of CMR as well as updates on the history, clinical indications, cost-effectiveness, role of artificial intelligence in CMR, and examples of common late gadolinium enhancement (LGE) patterns. This literature review was performed by searching the PubMed database for the most up-to-date literature regarding these topics. Relevant, less up-to-date articles, covering the history and physics of CMR, were also obtained from the PubMed database. Clinical indications for CMR include adult congenital heart disease, cardiac ischemia, cardiomyopathies, and heart failure. CMR has a projected cost-benefit ratio of 0.58, leading to potential savings for patients. Despite its utility, CMR has some drawbacks including long image processing times, large space requirements for equipment, and patient discomfort during imaging. Artificial intelligence-based algorithms can address some of these drawbacks by decreasing image processing times and may have reliable diagnostic capabilities. CMR is quickly rising as a high-resolution, non-invasive cardiac imaging modality with an increasing number of clinical indications. Thanks to technological advancements, especially in artificial intelligence, the benefits of CMR often outweigh its drawbacks.

## Introduction and background

Cardiovascular diseases (CVDs) remain the leading cause of death globally. In 2021, an estimated 17.9 million people died from CVDs, representing 32% of global deaths [1]. Early identification and intervention are vital in reducing mortality related to CVDs. Many imaging options are available for the non-invasive evaluation of CVD, including cardiac magnetic resonance imaging (CMR), computed tomography, echocardiography, and more. CMR can assess a wide range of cardiac characteristics in an unlimited number of planes but has logistical drawbacks related to housing equipment, interpreting images, and duration of image acquisition and processing [2,3]. In recent years, artificial intelligence (AI) has proven effective at mitigating some of these drawbacks by automating segments of the CMR imaging process [4]. This review will give an overview of the history, physics, clinical implications, pros, and cons of CMR.

CMR was first used in the 1970s to observe myocardial consumption of adenosine triphosphate, and other phosphate groups, using ^31^P spectroscopy. By the 1980s, the role of CMR expanded to use the T1- and T2-weighted relaxation rates of ^1^H protons to characterize the myocardium and assess pathologies such as myocardial edema. In the 1990s, CMR contrast agents were developed and used to assess cardiac vasculature, including the aorta, coronary arteries, peripheral arteries, and myocardial perfusion. This breakthrough was particularly helpful in evaluating myocardial scars [5]. Now, CMR is commonly used to investigate a range of cardiac pathologies, including cardiac ischemia, cardiomyopathies, heart failure, and more [3].

## Review

Physical foundations of CMR techniques

**Figure 1:**
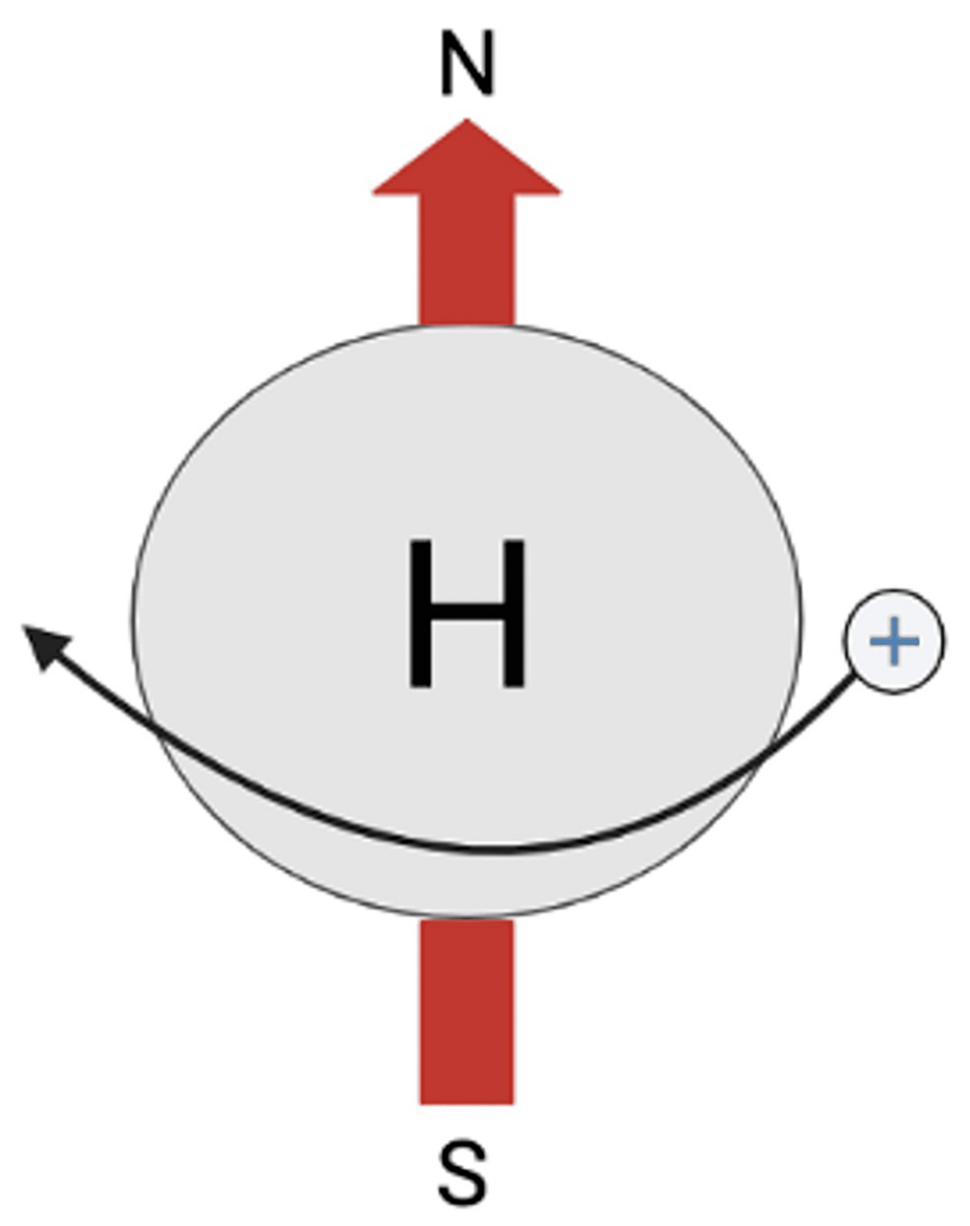
Hydrogen Atom and Its Magnetic Field This figure displays a rendition of a single hydrogen atom. There is a proton “+” spinning about its axis along a path depicted by the curved black arrow, creating a magnetic field. The vector of the magnetic field is depicted by a red arrow. The N and S poles describe the direction vector of the atom’s net magnetic field. N: North, S: South. Figure created by Mason Stoltzfus.

CMR is a specialized type of magnetic resonance imaging (MRI) that relies on the physical phenomenon of spin. Spin is a characteristic of some subatomic particles: all atoms with an odd number of neutrons or protons, such as the abundant proton Hydrogen ^1^H, exhibit spin. The charge and spin of a proton produce a magnetic field, allowing it to align with external magnetic fields like a simple magnet (Figure 1).

**Figure 2:**
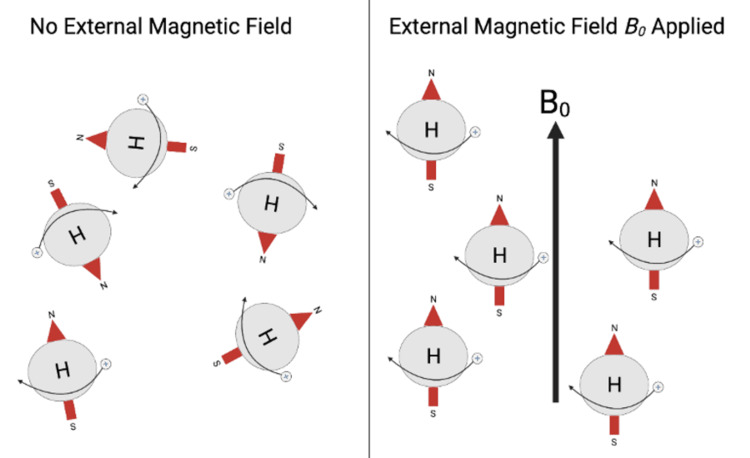
Hydrogen Atoms With and Without Application of an External Magnetic Field The left pane of the image, labeled "No External Magnetic Field", depicts hydrogen atoms with their individual magnetic fields, generated by spinning protons, in the absence of an external magnetic field. The right pane of the image, labeled "External Magnetic Field B_0_ Applied", depicts hydrogen atoms with their individual magnetic fields aligned due to the application of an external magnetic field, B_0_. N: North, S: South. Figure created by Mason Stoltzfus.

When protons in tissue are acted upon by an external magnetic field, such as the constant, homogeneous magnetic field B_0_, their longitudinal axes equilibrate parallel to a common vector of net magnetization [6] (Figure 2).

**Figure 3:**
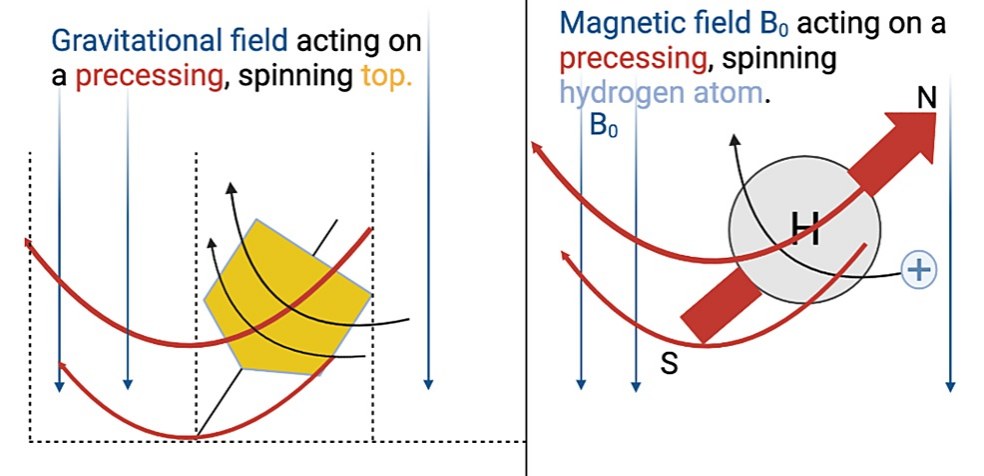
Depiction of Precession of a Spinning Top and Hydrogen Atom in Their Gravitational and Magnetic Fields, Respectively The left pane of the figure, labeled "Gravitation field acting on a precessing, spinning top", depicts a spinning top wobbling, or precessing, in a gravitational field after being tapped by an outside force (not depicted). The right side of the figure, labeled "Magnetic field B_0_ acting on a precessing, spinning hydrogen atom", displays an analogous depiction of a spinning hydrogen atom wobbling, or precessing, out of equilibrium in the magnetic field B_0_ after being disturbed by a second magnetic field, B_1_ (not depicted). N: North, S: South. Figure created by Mason Stoltzfus.

MRI systems rely on strong magnetic fields formed by the passage of electric current through coils of wire maintained at superconducting temperatures. In MRI systems, the subject lies supine inside a tube containing large coils of wire that wrap around the patient. Electric current passes through the coil, generating B_0_ along a vector parallel to the supine patient in a caudal-cranial or cranial-caudal orientation [7]. Nuclear magnetic resonance signals are generated by disturbing the magnetic field in equilibrium with B_0_ through additional exposure to an external magnetic field with a different direction vector, B_1_. Exposure to B_1_ causes the spinning protons to precess about their original longitudinal axis. This phenomenon is analogous to observing a top spinning parallel to Earth’s gravitational field, where the spinning top is analogous to the spinning proton, and Earth’s gravitational field is analogous to B_0_. If an external force with a vector different than gravity, analogous to B_1_, taps the top, forcing it out of alignment with the gravitational field, it wobbles or precesses about its transverse axis. Similarly, atoms in tissue aligned with B_0_ will precess when disturbed by B_1_ [6] (Figure 3).

**Figure 4:**
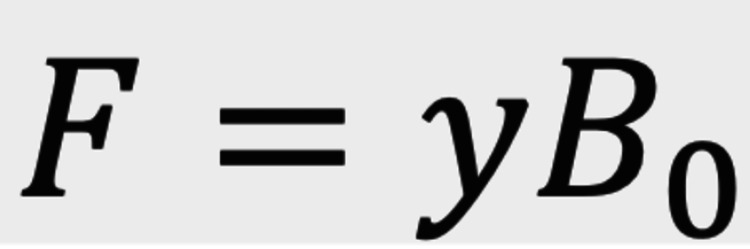
The Larmor Equation This figure displays the Larmor equation that is used to calculate the frequency of precession of a proton in a magnetic field, where *F *represents the frequency of precession, *y* represents the gyromagnetic ratio, and *B_0_* represents the strength of the homogeneous magnetic field acting on the proton. Figure created by Mason Stoltzfus.

The Larmor equation is used to determine the frequency of precession of various atoms in magnetic fields (Figure 4).

This frequency is used to time the efficient transfer of the B_1_ magnetic field, in the form of radiofrequency (RF) pulses, to the protons equilibrated with the B_0_ magnetic field. This efficient transfer of energy relies on the principle of resonance, which is well explained by pushing an object on a swing. When trying to push an object, such as a swinging pendulum, timing the push to resonate with the frequency of the swinging pendulum will provide the most efficient push. In a similar manner, for the efficient transfer of energy from B_1_ to precessing protons in tissue, the B_1_ RF pulses must resonate with the frequency of precession of the protons [7].

Following the disturbance of the homogeneous magnetic field B_0_ by B_1_, protons in the field precess about their transverse axes and the longitudinal equilibrium of the protons with B_0_ is temporarily lost. However, in a time-dependent manner, the magnetic fields of the protons slowly re-equilibrate with the ever-present, original B_0_ magnetic field vector. This phenomenon is known as T1 relaxation. Different tissues exhibit different time-dependent T1 relaxation rates, forming the basis for T1-weighted contrast in MRI [6]. In the setting of CMR, an increase in T1 relaxation rates is useful for identifying myocardial fibrosis, dilated cardiomyopathies, and low-flow, low-gradient aortic stenosis. Conversely, a decrease in T1 relaxation rates is associated with fatty deposits, such as glycosphingolipids associated with Anderson-Fabry disease [2,4].

Immediately following a B_1_ RF pulse, protons are no longer in equilibrium with the B_0_ magnetic field, but rather precessing in an identical fashion. These atoms precessing in sync, following exposure to an RF pulse, are described as being “in phase.” However, due to a variety of reasons, such as MRI systems’ limitations in generating a perfectly homogeneous magnetic field, protons do not precess identically indefinitely and gradually fall out of phase in a time-dependent manner. This phenomenon is analogous to tapping two spinning tops with identical external forces, B_1_, causing them to precess like the top in the left pane of Figure 3. The tops may initially wobble in unison, but the slightest differences in B_1_ forces applied due to experimental imperfection will eventually cause the tops to precess out of phase. This time-dependent decay of in-phase precessing of protons is referred to as T2 relaxation. Different tissues exhibit different time-dependent T2 relaxation rates, forming the basis for T2-weighted contrast in MRI [6]. CMR T2 mapping uses the principle of T2 decay to identify acute myocardial inflammation, which is common in the setting of acute myocarditis, myocardial infarction, sarcoidosis, cardiac transplant rejection, and toxicity due to chemotherapeutic agents [4].

Since tissue in B_0_ and B_1_ fields exhibit both T1 and T2 decay, it can be useful to minimize one type of decay and maximize the other. This creates T1- or T2-weighted imaging, where “weighted” refers to the decay effect being maximized [6].

Clinical indications of CMR

**Figure 5:**
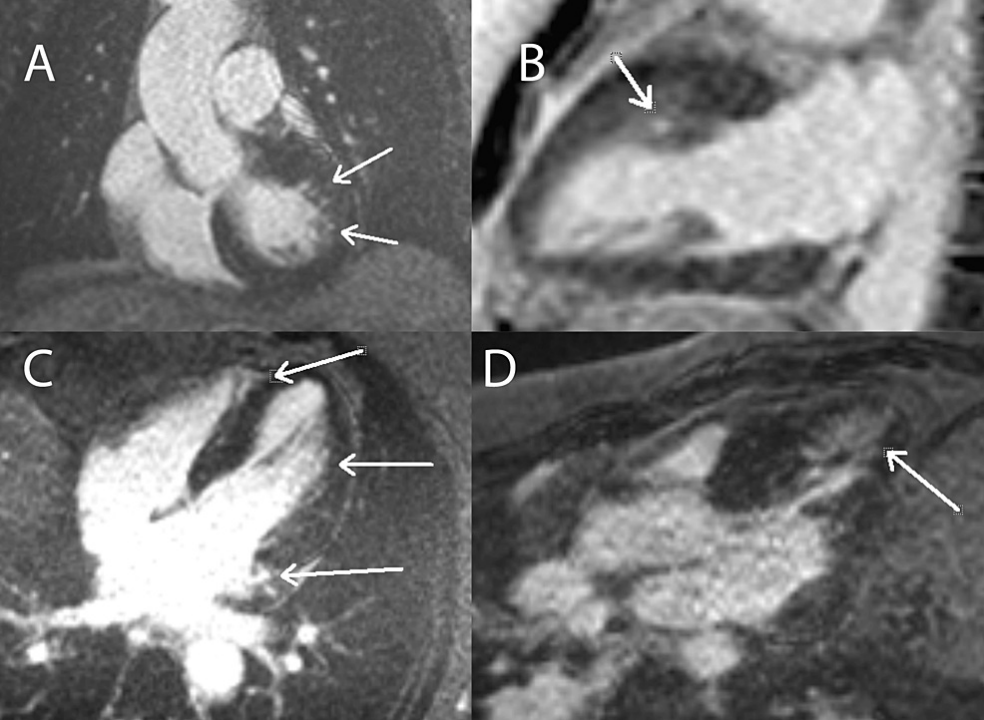
Hypertrophic Cardiomyopathy: Mid-Wall LGE Pattern This figure displays left ventricular outflow tract (pane A), two-chamber (pane B), four-chamber (pane C), and three-chamber (pane D) CMR sequence views from patients with hypertrophic cardiomyopathy. The arrows in all panes indicate patchy, mid-wall hyperintensities that resemble classic hypertrophic cardiomyopathy LGE patterns. LGE: late gadolinium enhancement. Figure created by Mason Stoltzfus.

CMR and its late gadolinium enhancement (LGE) sequences are indicated in the investigation of cardiac ischemia and infarction, amyloidosis, myocarditis, pericarditis and its sequela, and various cardiomyopathies. CMR can differentiate between etiologies of non-ischemic cardiomyopathies, including cardiac sarcoidosis, cardiac amyloidosis, acute myocarditis, hypertrophic cardiomyopathy (HCM), arrhythmogenic cardiomyopathy, and more by identifying LGE patterns in different tissues. In the setting of HCM, CMR can be used to quantify ventricular mass and detect fibrosis that is associated with a higher risk of cardiac death. For example, Figure 5* *displays multiple foci of patchy, mid-wall LGE affecting less than 5% of the myocardium, a pattern characteristic of hypertrophic and non-ischemic cardiomyopathies [2,3].

**Figure 6:**
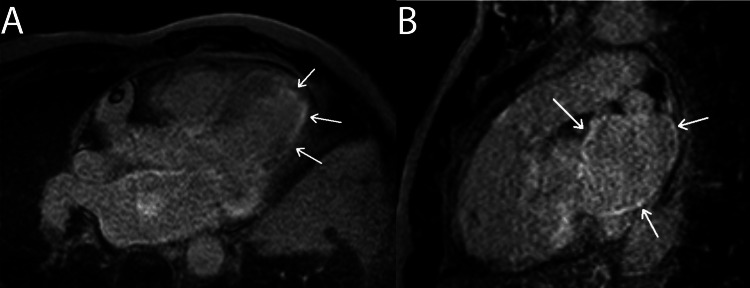
Cardiac Amyloidosis: Diffuse Subendocardial LGE Pattern This figure displays CMR sequences with three-chamber (A) and right ventricular outflow tract (B) views. The hyperintense regions indicated by arrows in panes A and B indicate diffuse subendocardial LGE, a classic finding in patients with cardiac amyloidosis. LGE: late gadolinium enhancement. Figure created by Mason Stoltzfus.

Figure 6* *demonstrates the classic diffuse subendocardial CMR LGE pattern that can be used to identify cardiac amyloidosis.

**Figure 7:**
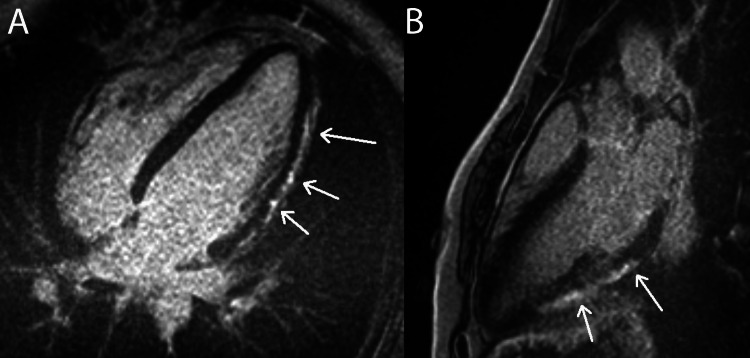
Myocarditis: Subepicardial LGE Pattern This figure displays both four-chamber (pane A) and three-chamber (pane B) CMR sequence views from a patient with myocarditis. The arrows in panes A and B highlight hyperintense subepicardial LGE patterns that spare the subendocardium, characteristic of myocarditis. In both panes, the LGE is most evident in the inferior left ventricle walls. LGE: late gadolinium enhancement. Figure created by Mason Stoltzfus.

**Figure 8:**
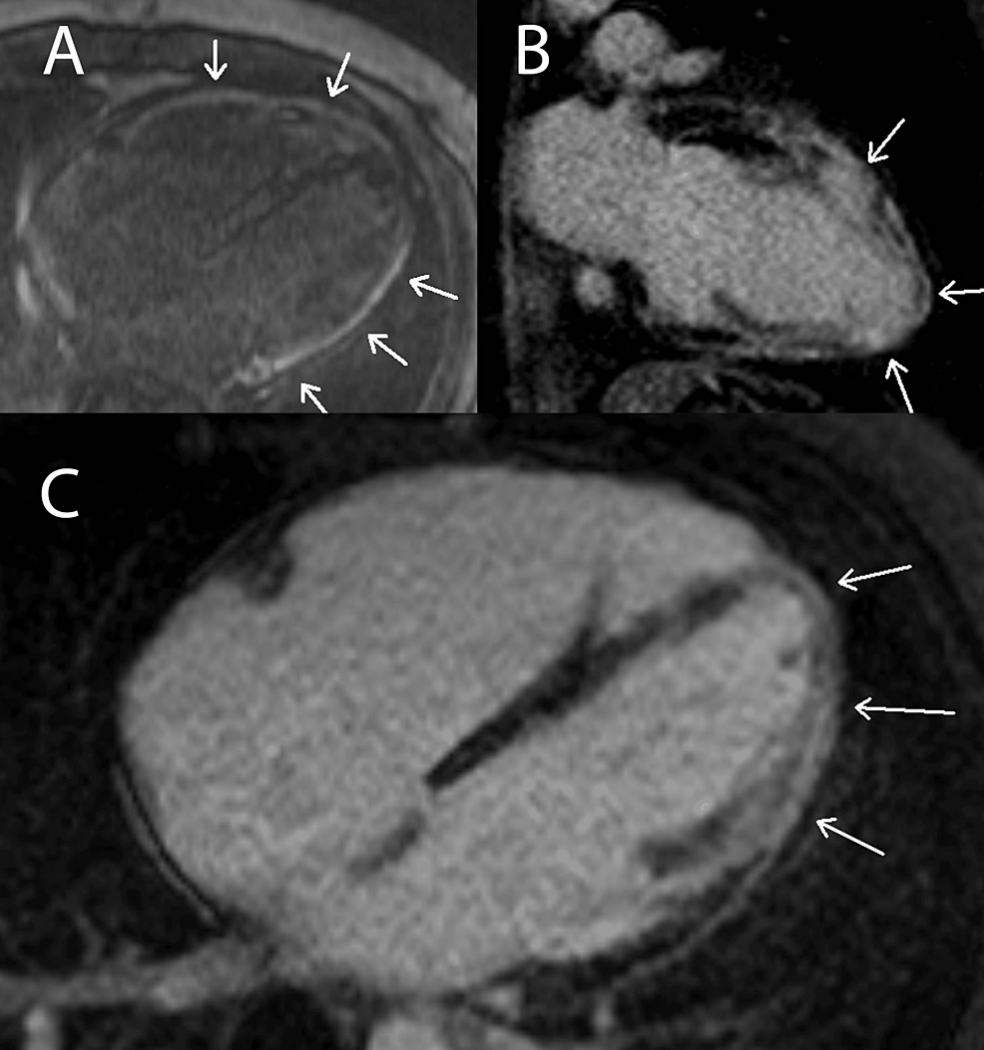
Myocarditis: Another Example of a Subepicardial LGE Pattern This figure displays both four-chamber (panes A and C) and two-chamber (pane B) CMR sequence views from a patient with myocarditis. The arrows in panes A, B, and C demonstrate hyperintense subepicardial LGE patterns that spare the subendocardium, characteristic of myocarditis. Panes A and B display subepicardial LGE along the mid and distal left ventricular wall. Panes B and C display apical LGE. Pane C highlights mid and distal right ventricle wall LGE. LGE: late gadolinium enhancement. Figure created by Mason Stoltzfus.

CMR is the non-invasive gold standard for diagnosing myocarditis and is capable of imaging acute and chronic tissue edema, hyperemia, and necrosis [8]. Figures 7, 8 include multiple examples of a classic myocarditis CMR LGE pattern: subepicardial LGE that spares the subendocardium.

**Figure 9:**
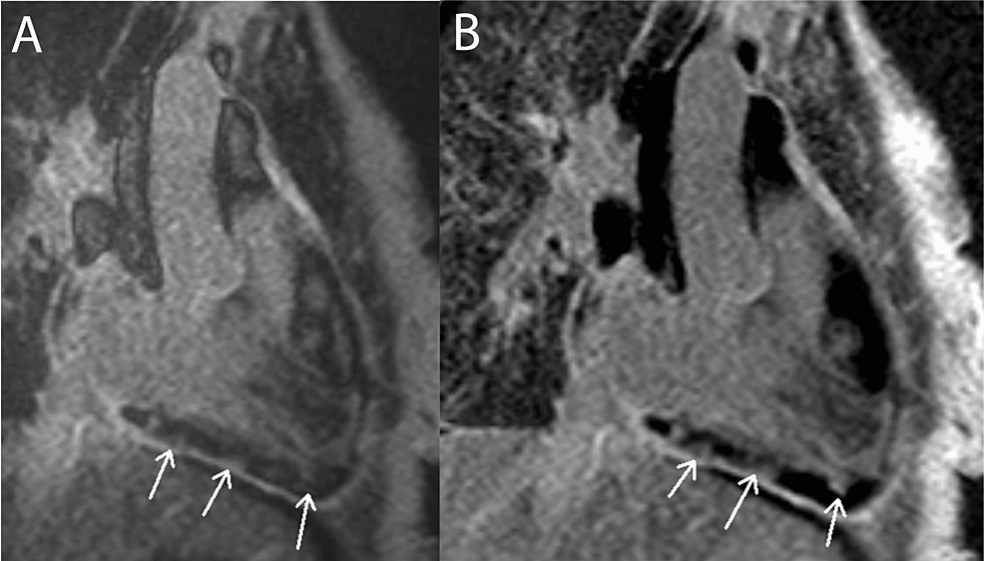
Pericarditis: Lace-Like LGE Pattern This figure displays right ventricular long-axis views from a patient with constrictive/restrictive pericarditis. Pane A displays LGE detected via magnitude inversion recovery and pane B with phase-sensitive inversion recovery. White arrows indicate hyperintense, lace-like pockets of LGE within a hypointense pericardial material collection, suggesting fibrous stranding throughout the collection. LGE: late gadolinium enhancement. Figure created by Mason Stoltzfus.

Figure 9* *exemplifies how CMR LGE can be used to detect fibrosis in a pericardial material collection in a patient with pericarditis.

Additionally, CMR can evaluate coronary artery disease (CAD) by identifying anomalous coronary arteries, scarring, and remodeling with different mapping and contrast enhancement [2]. CMR imaging in multiple planes, along with rest and stress perfusion tests, are helpful in detecting myocardial ischemia due to CAD [3]. In patients with stable chest pain suspected to have stable CAD, causing ischemia, CMR is the recommended imaging option. A large meta-analysis of CMR for the detection of CAD demonstrated a sensitivity of 89% (95% CI (88, 91)) and a specificity of 76% (95% CI (73, 78)) [9].

**Figure 10:**
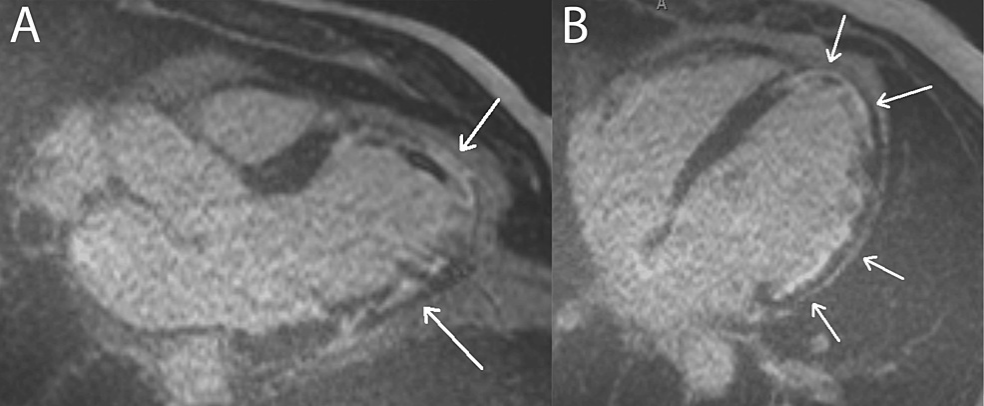
Myocardial Infarction: Diffuse Transmural LGE Pattern This figure displays both three-chamber (pane A) and four-chamber (pane B) CMR sequence views from a patient with left circumflex and mid-left anterior descending coronary artery occlusions leading to myocardial infarction in those distributions. The arrows in panes A and B demonstrate classic transmural and subendocardial hyperintense LGE due to transmural myocardial infarction. LGE: late gadolinium enhancement. Figure created by Mason Stoltzfus.

The CMR LGE sequences in Figure 10 are from a patient with myocardial infarction secondary to left circumflex and mid-left anterior descending coronary artery occlusions. In this case, the infarctions affect greater than 50% of the heart wall, especially at the apex. This led to the development of an aneurysmal apex and hypokinesis of the left heart wall, both of which were diagnosed via CMR. 

CMR T1 decay imaging is specifically indicated in the identification of glycosphingolipid deposition associated with Anderson-Fabry disease. This disease is characterized by glycosphingolipid deposition, followed by left ventricular hypertrophy (LVH), and ultimately fibrosis. These sphingolipids accumulate prior to LVH in 40-50% of patients. LGE and T1 mapping can even detect these sphingolipids and fibrosis in the absence of LVH and aid in diagnosing Anderson-Fabry disease [10]. The early diagnosis of this disease, using CMR, leads to early treatment with enzyme replacement therapy and improves clinical outcomes [11].

CMR T1 decay values also increase directly with myocardial water content, allowing the detection of myocardial edema, infarction, inflammation, fibrosis, myocarditis, and infiltration of amyloid, fatty deposits, or iron [12]. Up to 32% of iron overload cardiomyopathy cases have low native T1 relaxation rates (hyperintense on T1 sequences) and high T2 values. This allows for the early detection of this cardiomyopathy with CMR and improves survival with early chelation therapy treatment [12].

A commonly indicated CMR imaging modality, T1 mapping, generates images by corresponding tissue T1 relaxation rates at various points in tissue to individual pixels on an interface. These T1 pixel maps are useful for a quantifiable comparison of myocardial characteristics. T1 mapping is indicated in the imaging of cardiomyocyte intracellular and interstitial compartments when the use of potentially harmful gadolinium chelate-based contrast agents (GBCAs) is contraindicated [10,13]. T1 mapping is also helpful for quantifying the degree of myocardial fibrosis in patients with dilated cardiomyopathy by measuring extracellular volume (ECV). T1 mapping has even been found to have comparable efficacy to histological examination in the measurement of myocardial ECV [14]. Finally, T1 mapping can differentiate between viable and non-viable myocardium following acute myocardial infarctions with sensitivities of 79% and specificities of 79% (95% CI (76, 91)), as well as following chronic myocardial infarctions with sensitivities of 88% and specificities of 88% (95% CI (70, 100)), according to one study [15].

Pros of cardiac MRI

CMR assesses cardiac morphology, ventricular function, cardiac perfusion, vasculature, and metabolism of the heart by capturing high-resolution 3D images in unlimited planes, all without the use of ionizing radiation. There is a range of CMR pulse sequences and contrast options for creating personalized studies that evaluate multiple aspects of cardiac morphology and function. For example, perfusion scans with stress techniques and delayed contrast enhancement studies, such as LGE, can be performed in the same study to evaluate for myocardial ischemia, quantify infarction, and identify myocardial scarring. Furthermore, valvular planimetry using CMR cine images can be used to evaluate valvular disease, identify regurgitant jets, and quantify the aortic valve area. Valvular regurgitant fractions can also be calculated using CMR imaging with more accuracy than echocardiography [3]. While echocardiography is limited in its imaging planes, CMR has no such restriction, allowing for detailed assessment of cardiac wall thickness, the endocardium, specific structures, and more. CMR-obtained dynamic time-resolved (cine) images can be used to assess all stages of the cardiac cycle and are useful for assessing valvular function, anatomical abnormalities, and time-dependent cardiac chamber volumes [5]. CMR cine imaging can also be used with contrast to evaluate coronary artery perfusion and identify occlusive coronary artery disease. CMR can characterize cardiac tissue in ways no other cardiac imaging modality can: it can identify and even quantify myocardial edema, fibrosis, fatty deposition, iron deposition, thrombus deposition, and tumor growth and morphology. CMR angiography is available to map and measure vascular anatomy. CMR velocity encoding can be used to quantify the velocity of blood flow through vessels for valves and is helpful in evaluating intra-cardiac shunts, valvular regurgitation, and stenosis [2].

CMR contrast agents, including GBCAs, are safe for most patients, except for those who are pregnant or have end-stage renal disease [5]. The incidence of allergic reactions to GBCAs, about one in 10,000, is at least one order of magnitude lower than that of iodinated contrast agents used in computed tomography scans [16].

The cost of CMR, and all imaging, depends on multiple factors including the imaging facility, geographic location, identity of the third-party payer, and additional features such as contrast or stress imaging. According to the 2021 Medicare Physician Fee Schedule, the average cost of CMR for morphology and function without contrast in Manhattan, New York was $410.98. Although CMR may appear expensive at first glance, its cost-saving benefits make it worthwhile for many patients. In a retrospective study of 361 patients who received CMR imaging, Hegde et al. found that the average cost of CMR imaging was $2,382 per patient, but the cost-saving benefit was $4,690 per patient. The cost-benefit ratio for CMR in this study was calculated to be 0.508, and the average patient's net savings were $2,308. The savings were calculated by quantifying the savings from changes to patients’ care made from CMR images. Examples of cost-saving clinical impacts of CMR imaging include abortion of surgery, changes in medical therapy, and new diagnoses [17].

Cons of cardiac MRI

Despite its safety and utility, CMR has yet to become as prevalent as the mainstay cardiac imaging modalities of echocardiography, nuclear cardiology, and angiography. CMR is limited by the availability of equipment, trained operators, prolonged study times, and increased cost. CMR is performed using an MRI scanner equipped with CMR-specific hardware and software. CMR equipment requires more space than other imaging apparatus for separate rooms to house the equipment, controls, and power source [2]. This reality not only limits the portability of CMR but also its cost-effectiveness for low-volume healthcare centers. The equipment and facilities required for CMR also need to be staffed and maintained. A team of MRI physicists, MRI technicians, ancillary staff, and radiologists must be available to complete all steps of the CMR imaging process. Radiologists are required to time the administration of contrast, when required, and interpret the imaging results [18].

The strong magnetic fields generated by CMR also limit the items that can be present in both the operating room and the patient. Most ferromagnetic objects will accelerate toward the CMR coils, posing projectile hazards. Therefore, patients must be screened for implanted devices such as pacemakers, aneurysm clips, and any other ferromagnetic objects that may be acted upon by the CMR magnetic field [3]. While there are increasing numbers of MRI-compatible implantable devices, these devices can be the source of image artifacts. Prosthetic heart valves and stents are some of the common MRI-compatible devices that cause artifact during CMR imaging, in some cases making it difficult to visualize cardiac structures [19].

The duration of a CMR scan is typically 30 to 60 minutes but can be up to 2 hours, depending on its complexity [3,19]. This has implications on both the cost of the scan accrued through operator time and discomfort or inconvenience experienced by the patient [3]. While CMR can obtain cine images of the cardiac cycle, the process of obtaining these images requires more time than most imaging modalities and also the necessity for the patient to hold their breath for brief periods. This breath-holding requirement poses potential safety concerns for patients with cardiac and respiratory pathologies [2]. If a patient cannot hold their breath, such as those requiring sedation for imaging, this can lead to significant respiratory motion artifacts. This artifact can be overcome by some advanced techniques, but often leads to lower-quality CMR images being obtained from sedated patients [19].

The role of AI in cardiac MRI

The prolonged study times associated with CMR can be, in part, mitigated by AI-based algorithms. Multiple algorithms have been FDA-approved and are commercially available to aid in CMR image acquisition, image post-processing, image reconstruction, tracing to quantify cardiac mass and volumes, and risk stratification technologies. The use of AI with CMR decreases processing times and inter-observer variability while accelerating the completion of repetitive radiological tasks [4].

Deep learning, a subset of machine learning AI, employs layers of neural networks to use given input and output data to learn network parameters. Once taught network parameters, deep learning neural networks can independently perform complex tasks. The deeper the neural networks, or the more layers the system has, the more complex the tasks it can perform. Convolutional neural networks are an advanced class of deep learning that uses large datasets to amplify machine learning and processing of raw images [20].

One study employed a deep learning algorithm to effectively automatize the time-consuming process of segmenting both the cardiac ventricle’s endocardium and epicardium to measure ventricular function and wall mass [21]. Such automatization significantly decreases the human effort required to complete the time-consuming CMR segmentation process and reduces barriers to using CMR for more patients. Bai et al. used an AI algorithm to calculate cardiac ventricular mass and volumes for 5,000 patients using 93,500 database images. Their final CMR clinical measurement and segmentation accuracies, using a fully convolutional deep learning network, matched that of human experts [22].

## Conclusions

CMR is quickly gaining ground as the cost-effective gold standard of cardiac imaging. CMR is beneficial for the non-invasive diagnosis of adult cardiac ischemia, cardiomyopathies, heart failure, and more. CMR perfusion scans and LGE are also helpful in evaluating the myocardium following myocardial infarction and differentiating scar from viable tissue. 

While CMR is useful for imaging many cardiac pathologies, it has unique drawbacks. The image-capturing process can prove stressful for some patients with claustrophobia or difficulty holding their breath. Prolonged segmentation and processing times for CMR images are already being mitigated by automatization via AI algorithms. Space and staff requirements to house and maintain CMR systems are substantial.

Some AI algorithms are already proving effective in the automatic calculation of cardiac ventricular masses and volumes. Overall, CMR remains a strong option for cardiac imaging and will continue to become more usable as AI and CMR imaging technology advance.
